# Case of Remarkable Injury with Recovery

**Published:** 1890-12

**Authors:** E. A. Cobleigh

**Affiliations:** Dean and Professor of Theory and Practice of Medicine, in the Chattanooga Medical College, Chattanooga, Tennessee


### CASE OF REMARKABLE INJURY, WITH RECOVERY.

A Paper Read before the Tri-State Medical Society of Alabama, Georgia and Tennessee, October 15th, 1890, by E. A. COBLEIGH, M. D., Dean and Professor of Theory and Practice of Medicine, in the Chattanooga Medical College, Chattanooga, Tennessee.

GENTLEMEN OF THE TRI-STATE SOCIETY—I have the pleasuse of presenting to you to-day, though in a hastily written form, on account of press of other duties, the report of a case of injury which to me, at least, has seemed unique, both in the light of its final result and as to many of its manifestations while under my observation. With the report I hope to present the patient himself a little later.

Owing to the peculiarities of the case you will pardon me if I go into details somewhat at length, though I will be as brief as completeness of narration permits.

About three o'clock on the afternoon of August 6th, last, I received a telephone summons to go hurriedly to a manufacturing establishment in this city, where I was informed that a workman had just received a terrible injury, but the nature of which my excited informant at the phone could not state. Dr. Lesket was sitting in my office at the time and I invited him to accompany me, to which he readily assented. Taking my "emergency" satchel along, on the expectation that some operation might be necessary, we repaired with due despatch to the place. Before reaching our destination we could see several persons bearing a wounded man from one department of the works to another nearer the street, the victim being transported in a chair and in an almost upright sitting position. Having deposited their burden by the time of our arrival on a cot which was at hand, I

stripped him to the waist and undertook to place him in a recumbent posture, both for the comfort of the patient and to facilitate my own examination of his hurts. This was at once found to be utterly impossible, owing to the nature and degree of the injury sustained; every effort to materially lower the head and shoulders being attended with symptoms of collapse of an urgent nature. So he was propped in a semi-recumbent position, and the following history was obtained while we made our physical examination of the man:

An old well used for supplying the boilers with water had become inadequate for the purposes of the rapidly enlarging factory, and for a considerable period of time work had been going on in the way of deepening said well till it had reached sixty (60) feet below the surface. During the day a heavy steel drill had become so dull that another and smaller one had been substituted for it while the larger one went above for grinding on the power grindstone near the mouth of the shaft. This had been sufficiently sharpened, a loop of rope fastened around it and a fellow-workman was lowering it to the men below, when the noose loosened at a depth of about ten feet from the surface, slipped off and let the implement go dashing down on the men at the bottom with no warning worth mentioning, and it had struck the patient lying before me, after falling about forty-five or fifty feet.

At the bottom of the well, some of the men were holding the drill then in use, while Tony Houston, colored, the wounded fellow, was standing upright on a little rough platform about eighteen inches large which had been built to afford the striker an elevation from which to wield his sledge to the best advantage. It seems that they were awaiting the arrival of the larger tool from above, when the accident happened, and standing erect, but not all of them looking up as there was some dripping of water from the sides of the well necessitating heavy gum coats.

The implement went down sharp end first, and nearly or quite perpendicular, striking the victim unexpectedly, though the man at the top had shouted that the drill was falling. It struck the man on the back of the neck and plunged through the tissues to emerge from the right side of the chest, there protruding about eight inches, absolutely impaling him. Notwithstanding the force of the blow, Tony was not fairly knocked down, but was forced against the sides of the well in a sort of crouching position, without losing conscience for a moment even. He avers that he was standing absolutely erect at the time of the injury, though I surmise that he is mistaken in this matter, and I lean strongly to the opinion that he was very slightly stooping forward, perhaps from the warning given from above, as a man will instinctively cower when dreading that something will fall on him. With an exclamation of pain, and realizing what had happened, he stepped down from the platform, supporting himself against the side of the well, and called on a fellow-workman to pull out the drill. A very tall and stalwart negro (whom I saw at the time of my visit) undertook to do his bidding, standing on the same level with Tony, and finally using both hands for the purpose, but failed. So he mounted the platform and tried again by a steady pull, which did not budge the impaling instrument; and in his excitement, determined to get the thing out, he gave it that to-and-fro motion, with the powerful leverage of the long handle, which one often sees resorted to in pulling posts out of the ground. At this juncture the drill loosened, and he extracted it from above—just the reverse of its direction of entry.

Tony was now set in a bucket, very imperfectly fastened to the well-rope by a noose passed around him, and, holding himself mostly by his own efforts, was drawn to the surface, placed in the chair before mentioned, and conveyed to the work-room adjoining the office.

I will preface here by saying that Tony is a man of magnificent physique and splendid muscular development, having worked at hard and steady manual labor in this same factory for seven or eight years. He stands five feet, eleven inches high; is twenty-eight years old; scarcely ever ill a day in his life, though formerly given to occasionally "spreeing"; and weighs a hundred and eighty-five pounds.

My examination developed that the wound of entrance was situated one and a half inches to the right of the spinous process of the fifth cervical vertebra, just at the point where his neck began to broaden toward the shoulders, and the drill had only missed the spinal column by a hair's breadth. Passing downward and very slightly forward and to the right, leaving a rather smooth opening (oval perpendicularly) with somewhat inverted edges. It resembled the old-fashioned wounds of entrance of round shot, not very large—indeed not so immense as one would expect from the size of the wounding instrument, yet sufficiently so for the cervical muscles and fascia to show plainly in the wound, especially if it was forcibly opened. The shape of the wound made it close like a valve, yet air was entering and being expelled with a pink froth at nearly every respiratory effort, though there was no considerable hemorrhage. I first thought this air came from the air-passages of the lungs, but I have later formed the opinion that it was sucked in and out by the action of the diaphragm during the process of breathing.

From here the drill passed into the chest cavity between the scapula and the clavicle—at its very apex—without damage to either of these bones, impinging on the third and fourth ribs, which were both fractured from behind, directly in the line of the wound (evidently the fragments being parted as by a wedge while the drill was in situ), then passing down on the anterior surface of the fifth and sixth ribs without injury to either, and emerging by a great gaping and ragged wound, with much eversion of its edges, just at the inferior border of the latter rib and over the interspace below, its centre being at time of the examination two inches below, and one-and-a-half to the right of the nipple. There was only moderate bleeding from this wound, into the opening of which I readily introduced the tips of three fingers, and no air was escaping here. The skin and subcutaneous tissues seemed to be so absolutely deadened by the magnitude of the injury sustained as to have completely lost all their normal elasticity. I passed two fingers up the tract of the wound their full length, entering the pleural cavity with their tips under the broken ends of the lower fractured rib which could be distinctly felt. Everything felt torn and indefinite, the ends of the broken bone easily movable, but I was not able to satisfy myself by the touch with any degree of certainty whether the subjacent surface of the lung was injured or not, though I thought it was. From top to bottom of the wound, in its entire length, it measured in a direct line at that time fourteen and a half inches, and he must have had buried in his anatomy fourteen and a half inches of steel, an inch in diameter.

On withdrawal of the fingers the wound closed by collapse of its sides and prevented any profuse degree of hemorrhage externally. There was very intense pain and a marked degree of shock as shown mainly by the pulse, the mind remaining clear throughout. The integument, however, was quite clammy, and he complained of a great deal of chilliness, without any distinct rigor. There was very extreme rapidity and difficulty of respiration, some gasping, and I was strongly of the opinion that he was going to die in a short time, especially as I found the signs of depression increasing fast, the pulse losing all tone flickering, irregular, intermittent, and the mucuous surfaces blanching. I so expressed myself without hesitation to the employers, but added that in a few rare cases men had recovered miraculously from seemingly as desperate injuries.

He was at once given 1/100th gr. strychnia, 1/4th gr. morphia, and 1/100th gr. atropia hypodermatically. In fifteen minutes this was repeated, and twenty-five minutes later the strychnia was again resorted to. Very perceptible reaction resulted as shown by the improved state of pulse, and an hour and a half after I first got to him I had him removed to his home—one square distant—in the same chair previously in use. All motion was exquisite torture to him, especially the slightest moving of the right arm and neck. He was taken home in a semi-recumbent posture, with two men steadying his head in fixed position as they walked beside the bearers of the chair. Thus far the only treatment of the wounds by me consisted in applying pledgets of iodoform gauze over both openings, which readily adhered in place by the oozing from same. No alcoholics were given from the start, as I was anticipating hæmoptysis at any moment, and feared the least over-stimulation. There was only a slight (suppressed) cough and no spitting of blood took place, nor was there any emesis, though it was several times seriously threatened.

At his house he was disposed in nearly the same position previously resorted to, but put on a cot. Indeed he could not be laid down at all, every effort producing the most violent pain and alarming dyspnœa. Without medication and no other dressings I left him for an hour to meet another engagement. On my return he was resting as well as could be expected. Auscultation showed only shallow respiration in the upper part of the right side, almost no motion at all of the injured side of the thorax, solidity of the whole lung except the region of the upper lobe, and this locality afforded all kinds of coarse and fine moist râles. I should have stated at first that a previous auscultatory examination had shown the same state of affairs when the injury was received. There was no tendency to the least displacement of the ends of the broken ribs, and every movement of any part of the body, but especially of the head, neck, right arm and trunk, proved so very painful that I felt secure of no danger from this source, and I determined to leave him with nothing on the body save the gauze and a blanket to cover the surface. Morphine in sufficient doses to secure for him as reasonable an amount of comfort as possible was ordered, and he was left for the night in the care of two excellent colored attendants who had been furnished by his employers. Temperature now was normal and pulse of good volume but quick, owing to the loss of respiratory surface of the lung. Cough he could not because of the extreme suffering it produced, yet there was much inclination to do so, which he resisted with grim determination. Respiration of course continued rapid as at first.

I will here say that I had intended to present the original drill before you for your personal inspection, and have photographs of the instrument taken for publication, but it has been impossible for me to obtain possession of the drill and I can only give you the measurements of same which were accurately taken at the time. Its dimensions were found to be as follows: Six feet long; one inch in diameter, except at the sharpened extremity, where it was flattened out to a long diameter of one-and-a-half inches; it was an octagonal bar of solid and well tempered steel, weighing seventeen and three-quarter pounds. You can form a very accurate idea of the thing if you will picture it in your minds as being a sharpened "crow-bar" such as you see in every day use among masons and where heavy work is being done.

Next moning—the 7th—I found Tony had passed a very restless, wakeful night of suffering, but otherwise was not materially changed in condition from the night before. There had been but trifling hemorrhage from the lower opening, chest still full of blood-clot, râles as before, no cough or expectoration to speak of, and no blood in what he did spit up, but there persisted a very peculiar respiratory sound which I had never heard before, nor can I describe it with any degree of precision so as to give you a reasonable notion of what it sounded like. This was mingled with the other numerous chest râles, and the best description I can give is to liken it to the puff of the valve of a blacksmith's bellows—short, sharp, coarse, deep in tone, heard with both respiratory movements, but best and most pronounced at the beginning of expiration. It sounded more like air passing into the chest cavity from the larger tubes of the bronchi, yet I never could clearly make out that this was the case. No air was now passing through the openings made by the drill externally, and no signs of any emphysema of the tissues. Mobility of the entire right chest continued, the breathing being largely abdominal. Talking above a whisper was impossible, seemingly from a loss of power in the vocal cords or the muscles required for phonation. This latter continued for three or four days and disappeared gradually. Right arm was absolutely powerless, also continuing for many days, seeming to depend on the soreness of those muscles needed in the movements at the shoulder and neck, as well as the injury done to some of them directly or by the breaking of the ribs to which they happened to be attached. So pronounced was this condition that he would not allow the arm moved by another for some days, and afterwards he had it moved with great care and much complaint. Little appetite. Patient still compelled to half sit in one position, solely reclining on the back. Most of his complaint now was of the pain over the vicinity of the broken ends of ribs and in the injured cervical region. Arm only troublesome when moved. I now adjusted a bandage around whole of the thorax, using a stout towel for this purpose, drawing it pretty tight and fixing it with safety pins over the gauze which had been renewed. No straps were used as immobility of the right chest was perfect enough for all practical purposes, no tendency to any displacement of the broken ribs, and I wished to make free and frequent auscultation over the injured pleural cavity.

At three o'clock P. M. condition about the same, but felt more comfortable with bandage on than before. My impulse was from the very first to enlarge the wound of exit or otherwise provide for free drainage from the chest cavity, fully anticipating extensive empyema, but this was deferred mostly because I regarded the prognosis as desperately bad from the beginning. Pulse was now eighty-two, respiration forty, and temperature one hundred and three-fifths, showing reactionary fever. Appetite had improved. At nine P. M. pulse was eighty-six, respiration thirty-two, patient sleeping. Opiates, a placebo, and cleansing of the external wounds were the only remedial measure employed. Appetite had become craving during the day. Diet restricted to liquid foods in small quantity, frequently repeated, so as not to oppress the lung-action by distention of the stomach. Mainly confined to milk. Very thirsty.

On the 8th, at nine A. M., pulse ninety-six, respiration thirty-six, temperature one hundred. Rested well nearly all night. About the only complaint now is when he is moved in the least, and then his greatest solicitude is that his head and arm be carried along in exactly the same line with the rest of the body.

Skipping the record of the next two days, his pulse on the 12th was eighty-six, temperature a hundred and three-fifths. Other symptoms as before, but everything slowly improving. Râles, except the valve sound, all gone. Absorption of the clotted blood in the pleural cavity gradually taking place, and no special symptoms of interest. No fluctuation in chest and no signs of pus there. Wounds suppurating, but only a trifle.

At this stage the temperature fluctuated slightly for two or three days between 101½ and 99½. By the 17th it had fallen to normal, absorption had freed the entire surface of middle lobe of the lung and part of the lower lobe, so that respiration was taking place nicely in them. Pulse was still rather weak and rapid, and he was put on general tonics—mainly quinine and iron—with generous feeding. By the 20th he could be moved from the couch to a chair so as to have his bed "made up." Neck still stiff and side painful on motion of same, but otherwise not troublesome and arm now movable. Capable of lying in any position he wished except on the right side, and sleeping well nights. No cough worth mentioning at any time. Wounds granulating nicely under the simple dressing used, with very little suppuration, and he sticks to his bandage by his own expressed choice. About this time he was moved to the home of a sister, distant several blocks.

He now sat up more and more from day to day, was soon spending the most of his time in the shade of a tree in the yard, continued gradually but steadily improving up to the 30th, when both wounds were nearly well, the chest fully clear, and I discharged him as practically well himself, leaving him to wash and care for the rapidly granulating surfaces. His recovery seemed to be perfect, the ribs show no remaining callus, and so far as I have been able to determine there is no lung impairment He is now hearty and driving a dray for his livelihood, for a hardware firm.

At the time of the receipt of the injury the distance between the two wounds was, in a direct line, fourteen and a half inches. In a few days contraction of the integument decreased this, and now it measures barely thirteen inches. The scar of exit has finally fixed above the point occupied by the original wound at the time of the primary examination. Then it occupied the interspace below the sixth rib; now it is situated about over the rib, itself. Then it was two inches below the nipple; now only a half inch below.

I regard this case as one of remarkable recovery, fit to be recorded along with the celebrated "crow-bar" case of Maine, which was formerly classical in all the standard works on surgery, and the later case of abdominal perforation by a railroad coupling-link, which happened a few years ago in Kentucky. I consider it remarkable not only in itself and the recovery of the patient at all, but unique in many of its minute features. The escape from injury of any of the large vessels in the neck was most fortunate. So also as to the brachial plexus of nerves and the subclavian vessels. Had it happened to the other side of the neck this would probably not have been the case, because of the heart's relation to that side. The bare missing of the spinal column was another matter of peculiar luck for the patient. Probably no direction for perforation of the chest cavity with the same instrument could have been deliberately selected to better advantage for the victim.

Again, I regard the case as unique because of the total absence of an active or high grade of inflammation of the pleura or pulmonary tissue of the injured side, the lack of any empyema as subsequent to the admission of free quantities of air to the pleural cavity, and the freedom from serious cough or hæmoptysis. I could not tell from the shape of the wound which way the flat and broad edge of the drill entered. But at first I was fully of opinion that the drill had perforated the lung tissue, and that spitting of blood would occur within a few hours at the longest. This not happening I changed my views as to the perforation of lung, and came to the conclusion that the instrument passed down along the front surface of the lung, which it doubtless pushed aside, crowding it out of the way by the inherent pulmonary elasticity—which latter is proved by the compression possible and often observed from effusion into the pleural sac, this compression of course taking place at the expense of the air cells. That the surfaee of the lung was injured is not untenable I think, the drill probably ploughing a passage furrow of some depth along its track and hæmoptysis being prevented by the small size of the *torn* air-tubes, as well as by the firm and uniform compression by the surrounding profuse hemorrhage clotting about the whole lower part of the lung (as if it was in the embrace of a tourniquet), and which absolutely prevented the entrance of air into its interior. Of course this was not verified by an autopsy and must therefore remain solely as an hypothesis. But in any case it does seem that final expectoration of blood or pus would have probably shown itself, which was not the fact and which militates against the theory of injury of lung substance. Yet my clinical observation of the case led me very strongly (at the time he was under treatment) to the opinion that there was some degree of such injury. And the relative absence of confirmatory symptoms, such as are usual in like cases of damage to lung substance would only be in line with the peculiarity of this particular case in the total absence of pus in the pleural sac after admission of air there in large qauntities, as well as even the recovery of the patient at all after the receipt of so grave an accident.

NOTE.—The patient was present at the place of meeting of the Society for part of two days, during which time other papers had precedence. And in them the doctor read this paper. Tony had been compelled to go away and attend to his accustomed duties. So he was not finally exhibited after all. But he is now driving his dray in the city of Chattanooga, working at hard labor and heavy lifting for a wholesale hardware company.

During his illness he was seen by Drs. Rathnell and Drake, colleagues, of the gentleman who read the report of the case. Dr. Cobleigh presented a bar of steel before the meeting, of the exact diameter and shape of the original drill, except that it was not flattened and sharpened into a drill for actual use. He also exhibited a drawing on the check-board of the instrument itself so as to show the shape of its broad "drill-point."

The paper was read late in the session when there was a press of other matters crowding on the Society, and the discussion was necessarily brief, but the opinion was expressed that the case was truly unique and remarkable in all of its features."

